# Social-Psychological Factors in Food Consumption of Rural Residents: The Role of Perceived Need and Habit within the Theory of Planned Behavior

**DOI:** 10.3390/nu12041203

**Published:** 2020-04-24

**Authors:** Jiaqi Huang, Gerrit Antonides, Fengying Nie

**Affiliations:** 1 Agricultural Information Institute of Chinese Academy of Agricultural Sciences, Beijing 100081, China; niefengying@caas.cn; 2 Urban Economics Group, Department of Social Sciences, Wageningen University, 6706 KN Wageningen, The Netherlands; gerrit.antonides@wur.nl

**Keywords:** theory of planned behavior (TPB), perceived need, habit, food consumption, rural residents

## Abstract

To address the problem of malnutrition in poor rural areas of China, this study aims to examine the effects of social-psychological factors in food consumption of rural residents in poor counties of Southwest China. In addition, it investigates the role of perceived need and habit within the theory of planned behavior (TPB) in predicting food consumption. A survey with random sampling was conducted on rural residents (*n* = 424), and the theoretical frameworks of both the standard and extended TPB were applied for comparison purposes. Structural equation modeling was applied to test the relationships among constructs. Consumption of five food items was studied, respectively: meat, eggs, dairy, fish, and fruits. Results showed that incorporation of perceived need and habit substantially increased the explanatory power of the TPB, but these factors only had significant direct effects on intention rather than behavior. Perceived need and habit are stronger predictors of intention than any other TPB construct for consumption of all food items except for meat. We found indirect effects of the constructs in the extended TPB model on consumption to be different across food items. Practical implications to improve consumption of different food items were proposed accordingly.

## 1. Introduction

Eliminating hunger and malnutrition in all forms in the world by 2030 is a fundamental part of the Sustainable Development Goals of the United Nations. Micronutrient deficiency, as one form of malnutrition, is affecting more than two billion people in the world [1], and is particularly prevalent in poor, rural areas in developing countries [2,3]. A growing literature has documented that low dietary diversity is the main cause of micronutrient deficiency [4]. Hence, a varied diet is considered essential to decrease micronutrient deficiency and achieve positive health outcomes [5,6]. Many countries have developed and promoted national dietary guidelines and recommended consumption quantities of core food groups, such as grains, vegetables, fruit, meat, fish, eggs and dairy [7]. However, inadequate consumption of certain foods has been observed, particularly in developing countries [8,9,10]. China, although having greatly reduced its hunger population since the 1970s, is still home to 123.5 million undernourished people [10]. A survey in poor, rural counties in China in 2015 showed that as high as 99.4% of people consumed inadequate amounts of dairy and 93.4% consumed inadequate amounts of fish as compared with the lower limit of the daily intake level as recommended by Chinese dietary guidelines. The percentages for eggs, fruit, and meat were 79.3%, 73.2%, and 37.5%, respectively [11]. The observed high percentage of inadequate consumption is consistent with other studies showing that malnutrition in China is especially severe in poor, rural areas [12,13].

To improve dietary diversity and increase the consumption of target food groups, it is essential to know the determinants of people’s food choices and the magnitude of the effects of those determinants on consumption. Consumer food choices are complex and not only related with economic factors, but also with social and psychological influences [14]. However, food consumption studies of rural residents in developing countries have focused relatively often on economic factors such as prices, income, and market development [15], and have rarely covered social and psychological influences.

Social-psychological models are useful tools to analyze decision-making factors and processes, and thus informative to design interventions to change target behaviors into favorable directions [16]. Among those models, the theory of planned behavior (TPB) is popular and has been widely applied in predicting consumer intentions and behaviors in many domains [17], including food choice [18]. In brief, the TPB proposes that people’s behavior is predicted by intention and perceived behavioral control; intention, in turn, is predicted by attitudes, subjective norms, and perceived behavioral control. From systematic literature reviews of the TPB application to dietary behavior and food choice, the TPB predicted food consumption intentions and behaviors well [18,19,20]. The most recent systematic literature review on food choice shows that attitude was most strongly correlated with intention (r = 0.54), followed by perceived behavioral control (r = 0.42), and subjective norms (r = 0.37). Intention had a larger association (r = 0.45) with behavior than perceived behavioral control (r = 0.27) [20]. However, of the 43 related studies (see the list of studies in [20]), only seven were conducted in developing countries, and none were conducted among rural residents.

Although the TPB was proven to be valid in predicting food choice intentions and behavior, critical views exist concerning the constructs of the TPB. Notably, perceived need and habit are two constructs suggested to be added to the TPB to predict food choice and dietary behaviors [21,22]. The former indicates whether a consumer perceives a food item as necessary to consume, the latter indicates whether a person shows habitual behavior of consuming a food item. These two factors were not considered in the standard TPB but were shown to predict intentions quite well. However, each of these two factors were studied independently in a few studies [21,22,23,24,25,26], and were never included jointly in one study together with the TPB constructs. Moreover, most food-related studies including perceived need or habit stopped at the stage of predicting intention. To what extent perceived need and habit also affects behavior is not clear. 

This study aims to explore social-psychological factors of food consumption of rural residents in poor counties of Southwest China, with a focus on food consumption far below the dietary recommendation levels but with rich nutritious value, including consumption of dairy, fish, eggs, and fruits. Meat consumption, although not far below the recommendation level, is also studied for comparison. To the best of our knowledge, no similar study with a focus on rural residents has been conducted in China. This study is complementary to existing literature by adding evidence of the TPB application to food consumption of rural residents in developing countries, and by examining the roles of perceived need and habit together with the TPB in predicting food consumption intentions and behaviors. In addition, we aim to know why some people choose to consume more of a certain food item and others do not, and why some food items are less frequently consumed than other food items by comparing detailed TPB items for different types of food. This information, together with the estimated effects of the TPB constructs, will be helpful in designing interventions to improve the consumption of target food items. 

In the following sections, the theoretical framework, hypotheses and methods are described. This is followed by a description of the estimated effects of the TPB constructs on the intention and behavior of consuming each food item, and a comparison of differences in detailed TPB items. We conclude with a discussion of the role of habit and perceived need in the TPB and the policy implications to improve consumption of dairy, fish, eggs, and fruits.

## 2. Theoretical Framework

### 2.1. Theory of Planned Behavior

The theory of planned behavior (TPB), described by Ajzen [27,28,29], is now one of the most commonly used social-psychological models for understanding human behavior. The TPB proposes that a specific behavior is predicted by the intention to perform the behavior and perceived behavioral control, which reflects the capability of people to perform the behavior.

According to the TPB, intention is determined by three different kinds of beliefs and evaluations of these beliefs: behavioral beliefs, which refer to the perceived consequences of conducting the behavior and the evaluations of these consequences; normative beliefs, which refer to the extent to which other people or groups the person finds important expect the person to conduct the behavior, and the motivation to comply with those important people or groups; control beliefs, referring to the perceived presence of factors that can influence one’s capability to perform the behavior, and the perceived power to control these factors. 

All three kinds of beliefs are assumed to be readily accessible in memory, and then lead to the formation of three constructs which are *attitudes* towards the behavior (produced by behavioral beliefs), *subjective norms* (produced by normative beliefs), and *perceived behavioral control* (produced by control beliefs). The TPB predicts that if a person has more positive or favorable attitudes and subjective norms and stronger perceived control with respect to performing a behavior, the intention to perform that behavior will be stronger. In turn, in addition to perceived behavioral control, stronger intention predicts higher probability of performing the behavior [30]. 

Attitude reflects how people perceive and evaluate different attributes of a given behavior. As stated by the TPB, attitude (ATT) toward a behavior is proportional to the sum of each strength of the behavioral belief *i* (*b_i_*) multiplied by the subjective evaluation of that belief’s outcome (*e_i_*). For instance, for the behavior of drinking milk, people may have two main behavioral beliefs, one being that drinking milk is healthy, the other that milk is tasty. The strength of the belief that drinking milk is healthy may be stronger (weaker) than the strength of the belief of tastiness. The evaluation of the outcome of tastiness, however, could be higher (lower) than the evaluation of healthiness, thus people may think tastiness is more (less) important than healthiness when making a decision of drinking milk. The outcome of the product of terms is essentially an empirical assessment.

Subjective norms reflect the impact of social aspects on decision making. Subjective norm (SN) is proportional to the sum of each strength of the normative belief *j* (*n_j_*) associated with a given social referent multiplied by the motivation to comply (*m_j_*) with the referent in question. Still taking drinking milk as an example, a teenager may hear frequent advice from his/her mother that drinking milk is good for his/her growth, and the teenager’s motivation to comply with his/her mother’s advice may also be high. 

Perceived behavioral control reflects the easiness of performing a certain behavior. The easiness may be dependent on abilities (skills, knowledge, etc.), resources (money, time, etc.), obstacles, and so forth. Analogous to ATT and SN, perceived behavioral control (PBC) is proportional to the sum of each strength of the control belief *k* (*c_k_*) (belief that a control factor is present) multiplied by the perceived power to control that factor (*p_k_*).

### 2.2. Extensions of the TPB and Hypotheses

There are mainly two kinds of extensions of the TPB, one focused on specification or classification within one of the TPB constructs, the other aimed at including additional constructs to the TPB to increase the explained variance of the model.

The attitude construct may have both positive and negative components, making the resulting outcome ambivalent [31]. For example, eating chocolate is enjoyable, but unhealthy. Several studies suggested capturing both positive and negative aspects to construct the attitude, although they showed that more ambivalent attitudes generally were associated with smaller attitude–intention correlations [32,33]. Attitude has also been distinguished into an affective component and a cognitive component. The former represents how people feel emotionally (e.g., enjoyable or not), the latter relates more to the evaluation of a utilitarian outcome (e.g., healthy or not) [34]. Evidence on healthy eating showed that in general affective components have a larger effect on intention than cognitive components [34,35].

Subjective norms have been classified into social norms and personal norms. Social norms are related to expectations of third persons and personal norms reflect personal moral obligations or ethical concerns [18]. In the context of food choice, moral obligations usually refer to food decisions made for the health and well-being of other family members [21,35].

Perceived behavioral control mainly includes facilitating or interfering conditions. Empirically, Verbeke and Vackier [21] included past behavior and habits as constructs of perceived behavioral control but did not explain why to include them as PBC theoretically. 

Habit, however, has more often been discussed as a separate predictor in the TPB [36,37]. It is because the TPB has been found less predictive for less-deliberative-processing decisions or habitual behaviors [38]. In other words, including habit as a separate predictor in the TPB tends to increase the proportion of variance explained for habitual behaviors. In food related studies, Verbeke and Vackier [21] found that, compared with including habit as part of PBC, including habit as a separate regressor in the TPB increased explained variance from 30.8% to 52.0%. Moreover, habit is a significant and important predictor of both intention and behavior. Verbeke and Vackier [21] found the coefficient of habit to predict the intention of eating fish to be 0.635, and Russell et al. [36] found the coefficient of habit to predict behavior regarding food waste to be 0.650, larger than any other predictor. Some studies included past behavior, a relevant variable that is not the same as habit, in the TPB to predict food choice [22,39]. Wong and Mullan [39] applied the TPB to predict breakfast consumption and found that, after including past behavior, the predictive power of the TPB increased but the effect of intention diminished. However, Ajzen argues that habit differs from past behavior, reflecting the stability of a behavior and theoretically cannot influence intention and behavior [28]. In general, habit is supported as being a separate regressor in the TPB since presence of the habitual level may change the influence of other TPB constructs on intention and behavior [40]. 

Perceived need is another factor that has been considered for inclusion in the TPB as a separate predictor, especially in the domain of food choice. Paisley and Sparks [22] first introduced perceived need as an additional predictor, arguing that the TPB constructs do not include information on whether people see themselves in need of performing a certain behavior. It is possible that a person has a positive attitude, subjective norm, and perceived behavioral control towards performing a particular behavior, but perceives no need to do so. The addition of perceived need was found to explain a further 5% of the variance in intention to reduce fat intake [22], a further 6% and 11% of explained variance in intention to eat a low-fat diet and to eat five portions of fruit and vegetables per day, respectively [23], and a further 3% of the explained variance in intention to eat healthy [24]. Raats et al. [41] found perceived need to be the most important and independent predictor of intention to make dietary changes. Payne et al. [24] also found perceived need to be the most predictive of intention to eat healthy, but not predictive of healthy eating behavior. In general, existing studies show that perceived need is an important predictor of food-related intentions, but very few studied its role in predicting behavior. 

This study aims to explore social-psychological factors in the consumption of meat, eggs, dairy, fish, and fruits by rural residents. Consistent with the TPB and its extensions regarding specification, we included both affective and cognitive components in attitudes, and both social and personal norms in subjective norms. We expect that attitudes, subjective norms, and perceived behavioral control will predict intention, and intention and perceived behavioral control will predict behavior. Thus:

**Hypothesis** **1** **(H1)**.  *Attitudes towards consuming meat/eggs/dairy/fish/fruits are positively associated with intention to consume meat/eggs/dairy/fish/fruits*.

**Hypothesis** **2** **(H2)**.  *Subjective norms towards consuming meat/eggs/dairy/fish/fruits are positively associated with intention to consume meat/eggs/dairy/fish/fruits*.

**Hypothesis** **3** **(H3)**.  *Perceived behavioral control towards consuming meat/eggs/dairy/fish/fruits is positively associated with intention to consume meat/eggs/dairy/fish/fruits*.

**Hypothesis** **4** **(H4)**.  *Intention to consume meat/eggs/dairy/fish/fruits is positively associated with behavior of consuming meat/eggs/dairy/fish/fruits*.

**Hypothesis** **5** **(H5)**.  *Perceived behavioral control of consuming meat/eggs/dairy/fish/fruits is positively associated with behavior of consuming meat/eggs/dairy/fish/fruits*.

With regard to including habit into the TPB, we believe food consumption decisions are frequent and habitual. Therefore, we think habit is an important predictor. In addition, we found that previous studies including perceived need stopped at the phase of predicting intentions, not predicting behaviors. To our knowledge, no food-consumption-related research has extended the TPB by including both habit and perceived need as separate predictors. Thus, we will examine whether they predict both intention and behavior.

**Hypothesis** **6** **(H6)**.  *Perceived need towards consuming meat/eggs/dairy/fish/fruits is positively associated with intention to consume meat/eggs/dairy/fish/fruits*.

**Hypothesis** **7** **(H7)**.  *Habit towards consuming meat/eggs/dairy/fish/fruits is positively associated with intention to consume meat/eggs/dairy/fish/fruits*.

**Hypothesis** **8** **(H8)**.  *Perceived need towards consuming meat/eggs/dairy/fish/fruits is positively associated with behavior of consuming meat/eggs/dairy/fish/fruits*.

**Hypothesis** **9** **(H9)**.  *Habit towards consuming meat/eggs/dairy/fish/fruits is positively associated with behavior of consuming meat/eggs/dairy/fish/fruits*.

**Figure 1:**
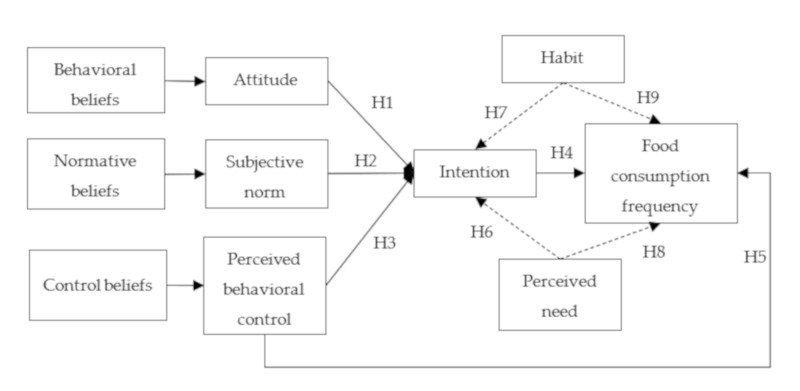
Specification of the extended theory of planned behavior (TPB) model and tested hypotheses.

In addition to testing the above nine hypotheses, summarized in Figure 1, we also aim to know how different people evaluate each detailed TPB item (belief strength and importance evaluation). We will compare people who consume meat/eggs/dairy/fish/fruits more frequently with those consuming less frequently and compare more frequently consumed food items with less frequently consumed food items. These comparisons are considered exploratory and we do not state hypotheses for them.

## 3. Method

### 3.1. Data Collection and Sample

We conducted a face-to-face household survey of 456 households in 76 villages of four poor counties of Yunnan and Guizhou Provinces in Southwest China in August of 2018. From the National Plan for Poverty Reduction between 2011 and 2020, the Chinese government has designated 592 national poor counties. The four sampled counties were selected from those 592 national poor counties based on their willingness to cooperate and high prevalence of small-scale farming. 

In each of the four counties, 19 villages were selected using the probability-proportional-to-size (PPS) method, and in each village six households were randomly selected. The survey included self-reported household information on food consumption frequency (in the previous seven days) and food consumption quantity (in the previous 30 days) for specific food items, household expenditure, income, and demographics. Well-trained enumerators asked the questions to the respondents and recorded their answers on electronic questionnaire equipment.

As part of the survey, a TPB questionnaire with regard to consumption of meat, eggs, dairy, fish, and fruits was presented. Each respondent was asked TPB questions with regard to only one food item. The food item was randomly selected by the enumerator through the random number generator on the electronic questionnaire equipment. After excluding 32 invalid responses (due to, e.g., missing answers) from the total sample of 456 participants, 424 valid responses to the TPB questionnaire were obtained, including 86 responses to the TPB questions for consuming meat, 92 for eggs, 85 for dairy, 83 for fish, and 78 for fruits. 

Table 1 shows the characteristics of the total sample, of which 64.86% were male and 74.29% were engaged in agriculture. The mean age was 52.33 years, and all respondents were adults above 18 years old. The mean household size was 3.50 persons. Nearly half the sample was from Guizhou province (51.88%), the other half from Yunnan Province. 

### 3.2. Measures

The measures from the TPB questionnaire with regard to consuming meat/eggs/dairy/fish/fruits will be described next. Internal reliability of the scales for each component was tested by Cronbach’s alpha. If Cronbach’s alpha was higher than 0.6, then the constructed component was considered reliable [21].

*Attitude*. The strengths of four belief attributes (*b_i_*) were measured on 5-point Likert scales, running from 1 (totally disagree) to 5 (totally agree), for each of the statements: “Eating meat/eggs/dairy/fish/fruits is healthy,” “Eating meat/eggs/dairy/fish/fruits is nutritious,” “Meat/eggs/dairy/fish/fruits tastes good,” “I am very satisfied when I am eating meat/eggs/dairy/fish/fruits.” The first two statements reflected people’s cognitive attitude components, while the latter two represented affective components. The evaluations of belief attributes (*e_i_*) were also measured on 5-point importance scales, running from 1 (totally unimportant) to 5 (very important), for each of the questions: “To what degree do you find the healthiness/nutrition/taste/satisfaction (asked in sequence) important when making a choice to eat meat/eggs/dairy/fish/fruits?” The responses of strength and evaluation of each belief attribute were multiplied (b_i*_e_i_), resulting in four scores for each respondent. Cronbach’s alphas for the four scores were 0.73 for meat, 0.63 for eggs, 0.79 for dairy, 0.82 for fish, and 0.87 for fruits. An overall attitude towards eating each of the five products (meat/eggs/dairy/fish/fruits) was calculated by taking the mean of the four scores:

ATT = ∑ *b_i_e_i_*/*I* (*i* = 1, …, *I*) (1)

where *I* is the relevant number of attributes comprising the attitude, and in this case, *I* = 4.

Subjective norms. Social norms and personal norms were both considered as components of subjective norms. The strengths of four normative beliefs (*n_j_*) were measured on 5-point Likert scales, for each of the statements: “My family thinks that I should eat meat/eggs/dairy/fish/fruits,” “Doctors think that I should eat meat/eggs/dairy/fish/fruits,” “To give my family a healthy diet, I buy meat/eggs/dairy/fish/fruits,” and “To give my family a nutritious diet, I buy meat/eggs/dairy/fish/fruits.” The former two were social norms and the latter two were personal norms. Motivation (*m_j_*) to comply with each normative belief was measured by asking for the level of importance (on 5-point Likert scales) of each normative belief when making a choice to consume meat/eggs/dairy/fish/fruits. Products of strength and motivation for each normative belief were created (n_j_*m_j_), and Cronbach’s alphas for the four products were 0.63 for meat, 0.72 for eggs, 0.73 for dairy, 0.84 for fish, and 0.86 for fruits. The mean of the four products of strength and motivation was calculated for each product for each respondent as the overall subjective norm:

SN = ∑ *n_j_m_j_*/*J* (*j* = 1, …, *J*) (2)

where *J* is the number of relevant social referents, and in this case, *J* = 4.

Perceived behavioral control. Affordability and accessibility of food items were considered as the main aspects of perceived behavioral control of food consumption of rural residents living in poor remote areas, respectively, indicating the beliefs about resources and obstacles that influence the decision of consuming a particular food item. The strengths of two control beliefs (*c_k_*) were measured on 5-point Likert scales for each of the statements: “Meat/eggs/dairy/fish/fruits is easily affordable for me,” and “Meat/eggs/dairy/fish/fruits is easily accessible for me.” The perceived importance (*p_k_*) of affordability and accessibility was measured by level of importance to them when making a choice to consume meat/eggs/dairy/fish/fruits. Control beliefs and perceived importance of affordability and accessibility were multiplied (c_k_*p_k_), and Cronbach’s alphas for the two scores were 0.69 for meat, 0.63 for eggs, 0.66 for dairy, 0.67 for fish, and 0.62 for fruits. The mean of the multiplications of control beliefs and perceived importance for affordability and accessibility was created for each product and for each respondent as the overall perceived behavioral control:

PBC = ∑ *c_k_p_k_*/*K* (*k* = 1, …, *K*) (3)

where *K* is the number of relevant attributes of perceived behavioral control, and in this case, *K* = 2.

Perceived need. Perceived need to eat meat/eggs/dairy/fish/fruits was measured by the response to the question: “To what extent do you feel that you need to eat meat/eggs/dairy/fish/fruits,” running from 1 (not at all) to 5 (to an extremely large extent).

Habit. Habits to eat meat/eggs/dairy/fish/fruits were measured by the response to the question: “To what extent do you agree that eating meat/eggs/dairy/fish/fruits is part of your eating habit?” running from 1 (totally disagree) to 5 (totally agree).

Intention. Intentions to eat meat/eggs/dairy/fish/fruits were measured by response to three statements: “I will consider to eat meat/eggs/dairy/fish/fruits in the next two weeks,” “I want to eat meat/eggs/dairy/fish/fruits in the next two weeks,” and “I plan to eat meat/eggs/dairy/fish/fruits in the next two weeks.” The response to each question was measured on a scale running from 1 (definitely not) to 5 (definitely). Cronbach’s alphas for the three questions were 0.94 for meat, 0.96 for eggs, 0.96 for dairy, 0.95 for fish, and 0.96 for fruits. The mean of the three intention scores was calculated for each product and for each respondent.

Behavior. The frequency of eating meat/eggs/dairy/fish/fruits was measured by how many days the respondent had eaten meat/eggs/dairy/fish/fruits in the previous seven days. The responses ranged from 0 to 7 days.

Statistics for all TPB items are presented in Table A1 in Appendix A.

### 3.3. Analysis

Data were analyzed using STATA 15.1. Relationships hypothesized by the TPB were tested through Structural Equation Modeling (SEM) for each food product separately [42]. Different from multiple separate regressions, SEM made it possible to study all of our hypothesized relationships in the TPB model in one analysis [43] and showed how some exogenous variables, for example, attitudes, subjective norms, and perceived behavioral control, were mediated by intention, allowing for indirect effects on behavior.

The fit of the TPB and its extended models was assessed by the following goodness-of-fit indices: chi-square and *p*-value, root mean square error of approximation (RMSEA), comparative fix index (CFI), the Tucker-Lewis Index (TLI), and standardized root mean square residual (SRMR). The model fit was considered good if chi-square was not statistically significant (*p*-value > 0.05), CFI and TLI were larger than 0.90 [44], and RMSEA and SRMR were less than 0.08 [45,46]. R^2^ of intention, behavior, and overall model were used to indicate the percentage of variance explained by the models.

*T*-tests were used to analyze the differences in each detailed belief strength and importance evaluation for each construct of attitude, subjective norm, perceived behavioral control, perceived need, and habit between groups of respondents with different consumption levels for a particular food item and between pairs of food items.

## 4. Results

### 4.1. Food Consumption Frequency

Table 2 shows the food consumption status of the study sample. The consumption frequency ranged from 0–7 days per week, and the frequency of meat consumption was the highest (5.70 days), followed by fruit (3.50 days), eggs (1.66 days), dairy (0.75 days), and fish (0.30 days). The consumption quantity per adult equivalent per day was converted from the total household consumption quantity in the previous 30 days for each food item. Comparing the actual consumption quantity per adult equivalent per day with the recommended lower limit for the consumption quantity of meat (40 g), eggs (40 g), dairy (300 g), fish (40 g), and fruit (200 g) by Chinese Food Pagoda [47], we were able to know whether each household had inadequate consumption for each food item. As high as 99% of the sample were short of consumption of dairy, followed by 94% for fish, 84% for eggs, 79% for fruit, and 13% for meat.

The statistics show that only meat consumption was not much lower than the recommended quantity in our sample. Consumption of dairy and fish particularly, together with eggs and fruit, were far below the recommended levels.

### 4.2. Differences in Belief Strength and Importance Evaluation by Consumption Frequency Level

A considerable percentage of respondents did not consume dairy, fish, eggs, and fruits in the seven days before the survey time. To explore the reasons for these differences in consumption frequency, we tested the differences in belief strength and importance evaluation for each belief item between groups of respondents whose consumption frequency was larger than zero and those whose consumption frequency equaled zero. Table 3 displays the results of the relevant *t*-tests. Since consumption frequency of meat was high and no zero frequency was observed, we only compare the differences for eggs, dairy, fish, and fruit.

Compared with subjects whose egg consumption frequency was zero, subjects who consumed eggs had significantly stronger beliefs that eating eggs was healthy, nutritious, tasty, and satisfactory. The differences in subjective norms and perceived behavioral control, however, were mainly due to the importance evaluations rather than belief strengths. Subjects who consumed eggs evaluated family’s and doctor’s opinions and preparing healthy meals for family as more important, and affordability as less important when making decisions on eating eggs. They also had significantly stronger perceived needs and habits. 

Subjects who consumed dairy had significantly stronger beliefs that consuming dairy was good for the family’s nutrition and affordable and had stronger perceived needs and habits as well. They also evaluated satisfaction, doctor’s opinion, and accessibility as more important.

Subjects who consumed fish had significantly stronger beliefs that eating fish was healthy, tasty, satisfactory, and affordable, and that family and doctors said they should eat fish. They also evaluated satisfaction and giving their family a healthy meal as more important. Habit and perceived need were significantly stronger for them.

As for subjects who consumed fruits, every facet of subjective norm was stronger, and they also evaluated tastiness as more important and had stronger habits and perceived need.

When comparing the magnitude of differences in each belief strength and importance evaluation, habit appeared to be the predictor with the largest differences for all four food items, followed by perceived need. For fish and fruit, the differences in habit and perceived need were both significant and larger than 1.00 on the 5-point Likert scales. In addition to habit and perceived need, beliefs of satisfaction showed large differences for eggs and fish; beliefs of family’s and doctor’s opinions showed large differences for fish and fruits; beliefs of giving family a nutritious meal showed large differences for dairy and fruit; beliefs of affordability showed large differences for dairy, fish, and fruit; beliefs of accessibility showed large differences only for fruit.

### 4.3. Differences in Opinions between Substitute Products

To gain some information about why people prefer to consume a certain food item rather than its substitute product, we compared the mean values of each belief strength and importance evaluation in each pair of food items (Table 4). One pair of substitutes was meat and fish, since they were both animal tissue containing animal protein and used as main dish for lunch or dinner. The other pair was eggs and dairy, since they were both products of animals containing animal protein and were mainly consumed for breakfast. Although the questions regarding meat and fish, egg and dairy were answered by different groups of subjects, the random sampling procedure ensured the validity of this comparison.

For the pair of meat and fish, Table 4 shows that subjects had significantly stronger intentions to eat meat than fish (Diff. = 1.58, *p* = 0.000). Among the differences in all belief strengths and importance evaluations, habit showed the largest difference (Diff. = 1.88, *p* = 0.000), followed by perceived need (Diff. = 1.12, *p* = 0.000), belief that eating meat/fish was good for the family’s health (Diff. = 0.56, *p* = 0.000) and nutrition (Diff. = 0.55, *p* = 0.000). Subjects also had stronger beliefs that eating meat was healthy, nutritious, accessible, affordable, and that their family and doctor told they should eat meat than for the same beliefs regarding eating fish. When making decisions on eating meat, subjects evaluated healthiness, taste, family and doctor’s opinion as being more important than when making decisions on eating fish.

For the pair of eggs and dairy, it was observed that subjects had significantly stronger intentions to consume eggs than dairy (Diff. = 0.98, *p* = 0.000). Habit again showed the largest difference (Diff. = 0.96, *p* = 0.000), followed by perceived need (Diff. = 0.63, *p* = 0.000), belief that it was satisfactory (Diff. = 0.65, *p* = 0.000) and tasty (Diff. = 0.62, *p* = 0.000). Subjects also had stronger beliefs that consuming eggs was healthy, nutritious, accessible, affordable, and good for the family’s health and nutrition. When making decisions on consuming eggs, subjects evaluated affordability as less important than when making decisions on eating fish.

### 4.4. Results of SEM Analysis

Table 5 shows the standardized results of the SEM analysis of the standard and extended TPB models for predicting intention and frequency of consuming meat, eggs, dairy, fish, and fruits. Compared with the standard TPB model, the extended TPB model included perceived need and habit as separate regressors, both for intention and frequency.

The standard and extended TPB model both fit the data perfectly for intention and frequency of eggs, dairy, and fish, as shown by the CFI of 1.000, TLI larger than 1.000, and RMSEA of 0.000. For meat and fruits, the goodness-of-fit statistics were less satisfactory but were still at an acceptable level regarding the SRMR and Chi-square (see Table 5).

In general, more variance of intentions was explained than of behaviors, in line with other food-choice-related studies [20]. Compared with the standard TPB model, the extended TPB model increased the explained variance of both intention and behavior, but more for intention. For example, for the estimation of dairy consumption, R^2^s of the intention and behavior equation were 0.193 and 0.120, respectively, in the standard TPB model, but increased to 0.628 and 0.161, respectively, in the extended TPB model. The same trend was also observed for the other food items.

#### 4.4.1. Prediction of Intentions

In the standard TPB models, attitudes significantly predicted intentions to consume meat (Coeff. = 0.311, *p* = 0.007), eggs (Coeff. = 0.341, *p* = 0.002), dairy (Coeff. = 0.383, *p* = 0.099), fish (Coeff. = 0.267, *p* = 0.017), and fruits (Coeff. = 0.279, *p* = 0.006). Therefore, Hypothesis 1 was supported for all five food items. Subjective norms, however, were only significant in predicting intentions to consume fish (Coeff. = 0.251, *p* = 0.031) and fruit (Coeff. = 0.401, *p* = 0.000), confirming Hypothesis 2 only for these two products. Perceived behavioral control, surprisingly, was not significant in predicting intentions to consume any of the food items. Hence, Hypothesis 3 was not confirmed.

In the extended TPB models, perceived need significantly predicted intention to consume all five food items (Meat: Coeff. = 0.460, *p* = 0.000; Eggs: Coeff. = 0.328, *p* = 0.001; Dairy: Coeff. = 0.270, *p* = 0.001; Fish: Coeff. = 0.398, *p* = 0.000; Fruit: Coeff. = 0.291, *p* = 0.003), supporting Hypothesis 6. Habit also showed significant effects on intention to consume eggs (Coeff. = 0.227, *p* = 0.016), dairy (Coeff. = 0.576, *p* = 0.000), fish (Coeff. = 0.342, *p* = 0.000), and fruits (Coeff. = 0.254, *p* = 0.005), but not meat. Hence, Hypothesis 7 was supported for four out of five food items. Moreover, perceived need and habit showed stronger effects on intention than attitudes and subjective norms.

Compared with the prediction in standard TPB models, the role of attitudes, subjective norms, and perceived behavioral control remained the same for intention to consume meat, eggs, dairy, and fruits in the extended TPB models, with the exception of fish. In the extended TPB model, perceived behavioral control became a significant predictor of intention to eat fish (Coeff. = 0.189, *p* = 0.012), whereas attitude and subjective norms became insignificant. A possible explanation is that for fish consumption, perceived need and habit were significantly correlated with attitude and subjective norms, but not with perceived behavioral control (see the correlation Table A2, Table A3, Table A4, Table A5 and Table A6 in Appendix A). Introducing perceived need and habit in the extended TPB model weakened the effects of attitudes and subjective norms, which is in line with the prior study that included perceived need to predict intention of healthy eating [24], and the study that included habit to predict intention to eat fish [21].

In summary, for most food items, perceived need, habit, and attitudes were significant predictors of intentions, and the effects of perceived need and habit were stronger than the effects of attitudes. As for the predictions by food item, intentions to eat fruit were predicted by perceived need, habit, subjective norms, and attitudes (ordered from the strongest to the weakest, similarly hereinafter); intentions to consume eggs and dairy were predicted by perceived need, habit, and attitudes, but for dairy, habit was the strongest predictor; intentions to eat fish were predicted by perceived need, habit, and perceived behavioral control in the extended TPB, but were predicted by attitudes and subjective norms in the standard TPB; intentions to eat meat were predicted by perceived need and attitudes.

#### 4.4.2. Prediction of Behaviors

The results from Table 5 showed that intentions significantly predicted consumption frequency of meat (Coeff. = 0.197, *p* = 0.057), eggs (Coeff. = 0.295, *p* = 0.002), fish (Coeff. = 0.438, *p* = 0.000), and fruits (Coeff. = 0.418, *p* = 0.000), supporting Hypothesis 4. Perceived behavioral control was only found to be significant in predicting the consumption frequency of meat (Coeff. = 0.227, *p* = 0.025) and dairy (Coeff. = 0.345, *p* = 0.000). Hence, Hypothesis 5 was only supported for meat and dairy, but not for eggs, fish, and fruits.

In the extended TPB models, perceived need and habit were not significant predictors of consumption frequency of any food items; therefore the direct effects stated in Hypotheses 8 and 9 were not confirmed.

However, as shown in Table 6, because of the effect of intentions, the indirect effects of attitude were evident on consumption frequency of eggs (Coeff. = 0.083, *p* = 0.039), fish (Coeff. = 0.041, *p* = 0.043), and fruits (Coeff. = 0.091, *p* = 0.032). The indirect effects of subjective norms were significant on consumption frequency of fish (Coeff. = 0.030, *p* = 0.060) and fruits (Coeff. = 0.119, *p* = 0.012). In the extended TPB, the indirect effects of attitude and subjective norms on consumption frequency were generally diminished. Attitudes only had significant indirect effects on consumption frequency of eggs. Subjective norms were not significantly predictive of consumption frequency of any food item. Instead, perceived need had significant indirect effects on consumption frequency of eggs (Coeff. = 0.472, *p* = 0.065), and fruits (Coeff. = 0.768, *p* = 0.081); habit had significant indirect effects on consumption frequency of fish (Coeff. = 0.320, *p* = 0.010).

To sum up, after including habit and perceived need in the TPB, the significant predictors of consumption frequency were intention and perceived behavioral control for meat; perceived need and attitude (indirect) for eggs; perceived behavioral control for dairy; habit (indirect) and intention for fish; and intention and perceived need (indirect) for fruits.

## 5. Discussion

### 5.1. Role of Perceived Need and Habit in the TPB

In this study, adding perceived need and habit in the TPB as predictors of intention and behavior substantially increased the explained variance of intention, but increased the explained variance of behavior by a small amount only. Moreover, perceived need and habit were only significant direct predictors of intention and not of behavior, which means habit and perceived need only influenced behavior indirectly via intention.

As for perceived need, this finding is consistent with the study of Payne et al., who found that perceived need was a significant and dominant predictor of intention to eat healthy, but not for eating behavior [24]. They interpreted this observation as perceived need only being relevant during the cognitive processing culminating in intention formation.

As for habit, our finding is consistent with Saba et al., who found that habit only predicted intention to drink milk but not behavior to drink milk [25]. However, Verbeke and Vackier found that habit had significant effects on both intention and behavior to eat fish [21], and so did Saba for a large variety of fat-containing food [26] and Faghih et al. for junk food consumption [48], which is different from our finding. Saba et al. claimed that the different and imprecise definition of habit may have contributed to different effect paths of habit in the TPB [26].

Another observation is that, apart from the prediction of meat consumption, perceived need and habit had larger effects on intention than other TPB constructs, and the inclusion of perceived need and habit diminished the effects of attitude and subjective norms on intention and the effects of intention on behavior. This observation is in line with the branch of studies including habit [25,38]. This effect was explained by the less conscious consideration of more habitual behavior. In other words, for frequently decided behaviors, people may apply limited cognitive processing, thus all constructs of the TPB requiring deliberation played a lesser role in making a decision, whereas habit played a larger role [49]. However, from our results, meat was the most frequently consumed food among all five food items, but the effect of habit was not significant in predicting intention, and the effects of other TPB constructs were also not diminished after including habit.

Instead, we turn to a possible alternative explanation: food involvement. Food involvement generally describes how deeply a person is involved in and thinks about food acquisition, preparation, cooking, eating, and disposal [50]. Verbeke and Vackier found that a lower food involvement level had a relatively strong impact of habit in fish consumption [21]. It is because food involvement is a better indicator to reflect cognitive complexity than frequency or how habitually a behavior is performed. A frequently performed behavior, like consuming meat in our study, may still be accompanied by extensive cognitive effort. For example, people may pay much attention to the quality of meat and spend time to select the piece of meat they want. This explanation is in line with the point that some behaviors, although containing automatic elements, are still reasoned in nature [40,51]. However, when looking at the results for dairy, which is the least frequently consumed product, the effect of habit was dominant. This means that for a rarely performed behavior, people may have too little information or experience to make cognitive efforts in decision making, and therefore, habit plays an important role: people who are not in the habit of consuming dairy usually also will not consume dairy. To sum up, the effect of habit on intention or behavior is not necessarily related to the habitual level of the behavior, but may be more related to the cognitive complexity or extensiveness of decision making. A less frequently performed behavior may have a stronger habit effect due to less information processed. 

As for the branch of studies including perceived need, although perceived need had significant and considerable influence on intention to eat fruit and vegetables, Povey et al. found that it did not change the effects of the TPB variables on intention [23]. However, in a study predicting healthy eating, inclusion of perceived need diminished the effects of cognitive attitude and subjective norm on intention, but not the effects of perceived behavioral control. This phenomenon was not explained by the authors [24].

Altogether, we found that both perceived need and habit predicted intentions well but did not predict behavior, and it is suggested that they be added to the TPB in similar future studies. However, the conceptualization of habit and perceived need and thus the link and interaction with other constructs in the TPB and the mechanism behind them needs to be studied further.

### 5.2. Practical Implications to Improve Consumption of Fish, Fruits, Dairy, and Eggs

To make suggestions for interventions to improve actual consumption of target food items, we need to focus on the significant predictors of behavior. In other words, if we found intention is not a significant predictor of behavior, then interventions targeting on all the other TPB constructs which influence intention may not be effective in changing behavior [52]. On the other hand, if intention is significant, the effects of TPB constructs on behavior may be smaller as compared with their effects on intention. This is because there may be other factors, e.g., institutional, situational or social, influencing behavior besides the TPB constructs.

For fish and fruit, consumption frequency is significantly predicted by intention, and in both cases perceived need is the strongest predictor of intention, followed by habit. Perceived need can be enhanced in two ways: making rural residents aware of the health benefits of eating fish and fruits; and making rural residents aware of their consumption deficit compared with the recommended level. Both these pieces of information can be included in existing nutrition education programs in poor rural areas. Habit, however, is difficult to change in the short term, especially for adults. The life course development of food choice shows that dietary habits mainly are formed in the early stages of life [18]. Hence, efforts to form healthy dietary habits are most effective for children and adolescents. Dietary education and meals at school are therefore essential to form healthy eating habits.

For fruit consumption, other significant predictors of intention are subjective norms and attitude. From the results in Table 3, we found that all facets of subjective norms showed significant differences between people who consumed fruits more frequently and those who consumed less frequently. Doctors’ and families’ suggestions on consuming fruits appeared to play a big role. Hence, promotion of the importance of consuming fruits could be included in local medical service training and in nutritional education programs. Thus, additional communication campaigns focusing on sharing dietary knowledge with people around may be effective. From the aspects of attitude, we know that the affective component, which is tastiness, played a large role. This is in line with other studies on healthy eating, which usually consider eating vegetables and fruits as a manner of healthy eating and that affective attitude is a stronger predictor of intention than cognitive attitude [24]. Advertisements or other promotion measures to make people feel that fruits are tasty may be effective in improving people’s intention to eat fruits.

For fish consumption, besides perceived need and habit, another significant predictor of intention was perceived behavioral control. Comparing people who consumed fish and who did not in the previous seven days of survey time, the significant difference in perceived behavioral control was mainly due to affordability. Comparing the differences in perceived behavioral control in consuming meat and fish, affordability and accessibility both were significant. Since the survey areas were mountain areas where fish supply was low, improving market access of fish and people’s purchase power to buy fish are essential measures to improve fish consumption in those areas.

For dairy consumption, intention was not a significant predictor of behavior but perceived behavioral control was. Like in the case for fish consumption, measures to improve accessibility and affordability of dairy both need to be undertaken.

Neither intention nor perceived behavioral control was a significant predictor of egg consumption. However, from Table 6 we knew that egg consumption was indirectly predicted by perceived need and attitude. Measures of both these predictors emphasize nutrition value and healthiness of eating eggs, and to diversify recipes of eggs in the nutrition education program may help to increase people’s overall attitude towards eating eggs. Measures to improve perceived need as described earlier, aiming to improve consumption of fish and fruits, can also be applied to the consumption of eggs.

Although for our sample the consumption levels of dairy and fish were extremely low and those of eggs and fruit were also relatively low, thus calling for an increase in consumption levels of these food items according to the Chinese dietary guidelines, it does not necessarily mean that more consumption would be better. The healthy reference diet recently proposed by the EAT-Lancet Commission, setting ranges of intakes for food groups to ensure human health [53], has recommended intake levels for dairy, fish, and eggs that are even lower than the lower limit of the recommended intake levels in Chinese dietary guidelines. The reference level of fruit intake is the same. The diverging guidelines exist because the EAT-Lancet Commission suggests a worldwide dietary transition aimed at increasing consumption of plant-based foods (like vegetables, fruits, whole grains, legumes, nuts, etc.) and decreasing consumption of animal-based foods (especially red meat). This dietary transition aims to deal with both diet-related human health issues and environmental problems. Combining the Chinese dietary guidelines and the EAT-Lancet Commission suggestions in designing interventions, information aiming for moderate consumption levels of food groups might be considered, as well as considering possible substitutes from plant-based foods if they are locally accessible.

### 5.3. Criticisms of the TPB

Despite the widespread use of the TPB regarding food choice and other health-related behaviors, there are still some criticisms to TPB. One branch of criticism focuses on the validity or the set-up of the TPB. One criticism is that all components and pathways in the TPB are considered rational without considering unconscious factors and influences on behavior [54]. For this criticism, Ajzen defended himself by explaining that the “planned” characteristics of the TPB do not imply rationality, and only meant that intention and behavior are consistently formed from readily accessible beliefs. However, beliefs can be informed poorly, which allows for and may reflect irrational processes [30]. Another criticism is that—although Ajzen claimed that background information such as demographics, emotions, personality traits, general values, etc. only influences beliefs and thus indirectly influences intention and behavior [30]—many studies showed that background information can have direct effects on behavior [55,56]. In addition, other factors, such as habit and perceived need in this study, self-identity [57], planning [58], etc. that can neither be grouped as background information nor as TPB components proved significant predictors of intention or behavior. That is why many extended TPB studies have been conducted.

The other branch of criticism is about the utility of the TPB; that is, whether the designed interventions based on the TPB results are useful and really lead to behavior change [55]. Some experimental studies showed failure of the TPB in causing behavior change [59,60]. However, recent studies seem to support the utility of the TPB. Hardeman et al. reviewed 30 papers and found half of the interventions based on the TPB were effective in changing intention, and two-thirds in changing behavior [61]. A more recent similar study on dietary behavior change showed that nine of eleven TPB based interventions resulted in dietary behavior change of adolescents and young adults [62].

One more query about the application of TPB in dietary change is that the TPB seems to be most predictive amongst the young, fit and affluent people [19], who differ substantially from the populations in which dietary behavior change is most needed. However, in our study we focused on the population aged over 18 years living in poor, rural areas in China that is most likely to suffer from malnutrition, and the explained variance of intention and behavior by the extended TPB was still generally high.

### 5.4. Limitations

Our study employed both a standard and an extended version of the TPB, which was estimated using structural equation modeling. Despite the extensive model, the main limitation of this study is that it is cross-sectional, prohibiting conclusions concerning causal effects. Although habits and perceived needs contributed to the explanation of food consumption intentions, the processes of habit formation and need development could not be traced in the cross-section. Hence, the utility of the interventions proposed from the results remains to be tested by more rigorous experiments. However, we think these exploratory associations at least offer important information on directions of efforts taken to improve consumption of target food items in poor, rural areas of China.

Another limitation is that although we had a total sample size of 424 respondents, they were randomly separated for questioning the consumption of five different food items, leading to relatively small samples to study the consumption of each food item. This small sample size restricted us from adding more background information such as demographics into the TPB model, acting as control variables and possibly leading to different estimation results.

### 5.5. Conclusions

In conclusion, this study provides support for the TPB in predicting food consumption of rural residents in poor counties of Southwest China, and to our knowledge, it is the first study to apply the TPB to study social psychological factors of food consumption for rural residents in China, and with a special focus on poor counties. The results are applicable in designing interventions aiming to improve dietary diversity and nutrition in poor rural counties of China where malnutrition is prevalent. Moreover, it shows that extending the TPB by including perceived need and habit substantially increased the explained variance of intention, and perceived need and habit only indirectly influenced behavior. For less frequently consumed food items, perceived need and habit are stronger predictors of intention than other constructs, and diminished effects of the standard TPB constructs were observed after including perceived need and habit. For more frequently consumed foods like meat, habit has no significant effect on intention, and no diminished effects of the standard TPB constructs were observed. This shows that less frequently performed behavior may have stronger habit effects on intention due to less information needing to be processed and cognitively analyzed. This explanation is different from other studies which consider habit only playing an important role in predicting habitual behaviors. Our study suggests that in order to increase dietary diversity by promoting less frequently consumed food, stimulating perceived needs and developing habits may be more important than strengthening attitudes, social norms and perceived behavioral control.

## Figures and Tables

**Table 1:** Characteristics of the study sample (*n* = 424).

Gender	%
Male	64.86
Female	35.14
Engaged in agriculture	74.29
Age	52.33 (12.66)
Household size	3.50 (1.53)
Province, County	%
Guizhou Province	51.88
Pan County	25.94
Zhengan County	25.94
Yunnan Province	48.11
Wuding County	23.35
Huize County	24.76

Note: standard deviations in parentheses.

**Table 2:** Food consumption status of the study sample (*n* = 424).

	Frequency (Day)		Consumption (g/Adult Equivalent/Day)		Inadequate Consumption (%)	
	M	SD	M	SD	M	SD
Meat	5.70	1.91	88.20	108.79	0.13	0.33
Eggs	1.66	1.98	22.78	41.25	0.84	0.36
Dairy	0.75	2.03	11.34	40.11	0.99	0.07
Fish	0.30	1.08	8.79	19.12	0.94	0.24
Fruit	3.50	2.63	131.11	139.36	0.79	0.40

**Table 3:** Differences in belief strength and importance evaluation by consumption frequency level.

	Eggs			Dairy			Fish			Fruit		
	Freq > 0	Freq = 0		Freq > 0	Freq = 0		Freq > 0	Freq = 0		Freq > 0	Freq = 0	
	*n* = 52	*n* = 40		*n* = 16	*n* = 69		*n* = 18	*n* = 65		*n* = 59	*n* = 19	
	Mean 1	Mean 2	Diff.	Mean 1	Mean 2	Diff.	Mean 1	Mean 2	Diff.	Mean 1	Mean 2	Diff.
**Intention**	3.79	2.87	0.93***	2.67	2.35	0.32	3.65	2.24	1.41***	4.12	2.89	1.23***
**ATT**	15.23	14.22	1.00**	13.11	13.33	−0.22	16.17	13.97	2.20***	16.44	14.67	1.77*
att1	17.15	15.75	1.40*	14.94	15.36	−0.42	17.00	15.02	1.98**	18.07	16.32	1.75
att1_str	4.04	3.75	0.29**	3.56	3.59	−0.03	4.00	3.66	0.34 *	4.15	3.89	0.26
att1_imp	4.23	4.17	0.06	4.25	4.26	−0.01	4.22	4.08	0.15	4.31	4.16	0.15
att2	16.23	15.05	1.18*	15.19	14.87	0.32	16.22	14.75	1.47*	16.80	14.95	1.85 *
att2_str	4.04	3.77	0.26***	3.81	3.75	0.06	3.94	3.77	0.18	4.05	3.79	0.26
att2_imp	4.02	3.98	0.04	4.00	3.94	0.06	4.11	3.89	0.22*	4.12	3.95	0.17
att3	14.52	13.70	0.82	11.94	12.26	−0.32	15.39	13.31	2.08**	15.97	13.79	2.18**
att3_str	3.85	3.52	0.32**	2.94	3.17	−0.24	3.89	3.55	0.34*	3.95	3.68	0.26
att3_imp	3.77	3.90	−0.13	4.13	3.88	0.24	3.94	3.77	0.18	4.00	3.74	0.26*
att4	13.00	12.40	0.60	10.38	10.83	−0.45	16.06	12.78	3.27***	14.93	13.63	1.30
att4_str	3.69	3.27	0.42***	2.63	2.96	−0.33	4.00	3.49	0.51 **	3.78	3.63	0.15
att4_imp	3.52	3.83	−0.31**	3.94	3.59	0.34 *	4.00	3.68	0.32 *	3.88	3.74	0.14
**SN**	14.97	13.86	1.11*	14.09	13.34	0.75	14.82	12.70	2.12**	17.03	13.09	3.94***
sn1	14.19	12.75	1.44*	12.25	12.35	−0.10	14.72	11.92	2.80**	16.47	12.11	4.37***
sn1_str	3.44	3.33	0.12	3.25	2.99	0.26	3.72	3.02	0.71***	3.98	3.26	0.72***
sn1_imp	4.12	3.85	0.27**	3.81	4.09	−0.27	3.89	3.89	0.00	4.10	3.68	0.42**
sn2	13.90	12.50	1.40	11.69	12.91	−1.23	14.39	12.20	2.19	17.19	11.21	5.98***
sn2_str	3.25	3.15	0.10	3.06	3.06	0.00	3.50	3.06	0.44*	4.03	2.89	1.14***
sn2_imp	4.21	3.95	0.26*	3.75	4.19	−0.44**	4.06	3.94	0.12	4.22	3.89	0.33*
sn3	15.96	15.20	0.76	16.31	14.33	1.98	15.56	13.29	2.26*	17.22	14.58	2.64**
sn3_str	3.85	3.77	0.07	3.75	3.42	0.33	3.72	3.37	0.35	4.07	3.68	0.38**
sn3_imp	4.13	3.98	0.16*	4.31	4.16	0.15	4.17	3.88	0.29*	4.19	3.95	0.24*
sn4	15.96	15.20	0.76	16.31	14.33	1.98	15.56	13.29	2.26*	17.22	14.58	2.64**
sn4_str	3.83	3.75	0.08	3.75	3.33	0.42*	3.61	3.43	0.18	4.08	3.68	0.40**
sn4_imp	4.10	3.95	0.15	4.25	4.07	0.18	4.00	3.85	0.15	4.17	3.89	0.27**
**PBC**	14.29	14.16	0.13	16.38	13.20	3.17***	14.44	13.34	1.11	15.93	12.16	3.77***
pbc1	13.92	14.57	−0.65	16.56	13.06	3.50***	14.50	12.95	1.55	15.66	12.42	3.24***
pbc1_str	3.88	3.75	0.13	3.88	3.23	0.64**	3.89	3.42	0.47*	3.90	3.26	0.64***
pbc1_imp	3.62	3.95	−0.33**	4.31	4.06	0.25	3.72	3.88	−0.15	4.02	3.84	0.17
pbc2	14.65	13.75	0.90	16.19	13.35	2.84**	14.39	13.72	0.67	16.20	11.89	4.31***
pbc2_str	3.98	3.75	0.23	3.88	3.49	0.38	3.78	3.54	0.24	4.02	3.26	0.75***
pbc2_imp	3.65	3.67	−0.02	4.19	3.80	0.39*	3.89	3.89	0.00	3.98	3.68	0.30
**PN**	3.79	3.15	0.64***	3.25	2.80	0.45*	3.83	2.72	1.11***	4.10	3.11	1.00***
**HBT**	3.50	2.65	0.85***	2.69	2.01	0.67**	3.44	2.09	1.35***	3.78	2.53	1.25***

*p < 0.1, ** *p* < 0.05, *** *p* < 0.01. Because of the moderate sample sizes, we also considered marginally significant coefficients with *p* = *0*.10 (similarly hereafter). “Freq = 0” means consumption frequency is zero. INT = intention, PBC = perceived behavioral control, HBT = habit, PN = perceived need, ATT = attitudes, and SN = subjective norm. “_str” means strength of belief, “_imp” means evaluation of importance. att1 = healthy, att2 = nutritious, att3 = tasty, att4 = satisfactory; sn1 = family’s opinion, sn2 = doctor’s opinion, sn3 = give family a healthy diet, sn4 = give family a nutritious diet; pbc1 = affordability, pbc2 = accessibility.

**Table 4:** Differences in belief strength and importance evaluation by food pairs.

	Meat (*n* = 86)	Fish (*n* = 83)	Diff.	Egg (*n* = 92)	Dairy (*n* = 85)	Diff.
**Intention**	4.12	2.55	1.58***	3.39	2.41	0.98***
**ATT**	15.92	14.44	1.48***	14.79	13.29	1.50***
att1_str	3.83	3.73	0.09	3.91	3.59	0.32***
att1_imp	4.31	4.11	0.21***	4.17	4.26	−0.08
att2_str	3.86	3.81	0.05	3.92	3.76	0.16*
att2_imp	3.97	3.94	0.03	3.97	3.95	0.01
att3_str	4.01	3.59	0.42***	3.67	3.06	0.62***
att3_imp	3.95	3.81	0.15 *	3.70	3.89	−0.20
att4_str	3.92	3.57	0.35 **	3.48	2.82	0.65***
att4_imp	3.88	3.75	0.14	3.52	3.55	−0.03
**SN**	15.26	13.16	2.10***	14.49	13.48	1.00**
sn1_str	3.59	3.17	0.42***	3.36	3.04	0.32**
sn1_imp	4.09	3.86	0.24**	3.97	4.04	−0.07
sn2_str	3.22	3.16	0.06	3.17	3.06	0.12
sn2_imp	4.09	3.89	0.20*	4.07	4.11	−0.04
sn3_str	3.97	3.41	0.56***	3.78	3.45	0.34***
sn3_imp	4.03	3.87	0.17	4.03	4.15	−0.12
sn4_str	3.99	3.43	0.55***	3.76	3.34	0.42***
sn4_imp	4.01	3.84	0.17	4.00	4.07	−0.07
**PBC**	14.88	13.58	1.30**	14.23	13.80	0.43
pbc1_str	3.78	3.52	0.26*	3.79	3.35	0.4***
pbc1_imp	3.86	3.81	0.05	3.73	4.07	−0.34***
pbc2_str	3.95	3.59	0.36***	3.85	3.53	0.32**
pbc2_imp	3.71	3.86	−0.15	3.63	3.84	−0.20
**PN**	4.08	2.96	1.12***	3.51	2.88	0.63***
**HBT**	4.27	2.39	1.88***	3.10	2.14	0.96***

* *p* < 0.1, ** *p* < 0.05, *** *p* < 0.01. INT = intention, PBC = perceived behavioral control, HBT = habit, PN = perceived need, ATT = attitudes, and SN = subjective norm. “_str” means strength of belief, “_imp” means evaluation of importance. att1 = healthy, att2 = nutritious, att3 = tasty, att4 = satisfactory; sn1 = family’s opinion, sn2 = doctor’s opinion, sn3 = give family a healthy diet, sn4 = give family a nutritious diet; pbc1 = affordability, pbc2 = accessibility.

**Table 5:** Structural equation modeling (SEM) analysis results of standard and extended TPB models for frequency and intention of consuming meat, eggs, dairy, fish, and fruits.

	Standardized Coeff. (Standard TPB)					Standardized Coeff. (Extended TPB)				
	Meat	Eggs	Dairy	Fish	Fruit	Meat	Eggs	Dairy	Fish	Fruit
Frequency										
INT	0.197 *	0.295 ***	0.023	0.438 ***	0.418 ***	0.231 *	0.192	−0.228	0.288 *	0.255 *
PBC	0.227 **	0.067	0.345 ***	−0.027	0.149	0.230 **	0.074	0.307 ***	−0.015	0.113
HBT						0.101	0.046	0.205	0.202	0.090
PN						−0.139	0.152	0.170	0.035	0.168
Constant	1.172	−0.439	−0.856	−0.584	−1.105	1.222	−0.95	−1.079	−0.856	−1.293
Intention										
PBC	0.107	−0.011	−0.016	0.143	0.160	−0.107	0.041	−0.080	0.189 **	0.061
ATT	0.311 ***	0.341 ***	0.383 ***	0.267 **	0.279 ***	0.346 ***	0.206 *	0.136 *	0.073	0.154 *
SN	0.143	0.136	0.107	0.251 **	0.401 ***	−0.076	0.061	−0.045	0.058	0.242 **
HBT						0.165	0.227 **	0.576 ***	0.342 ***	0.254 ***
PN						0.460 ***	0.328 ***	0.270 ***	0.398 ***	0.291 ***
Constant	3.142	0.420	0.320	−0.199	0.713	0.695	−0.665	0.179	−0.854	0.261
R^2^ (Freq)	0.114	0.096	0.120	0.186	0.265	0.126	0.118	0.161	0.213	0.288
R^2^ (INT)	0.206	0.179	0.193	0.275	0.538	0.438	0.384	0.628	0.578	0.677
R^2^ (Overall)	0.246	0.183	0.289	0.275	0.547	0.474	0.402	0.688	0.592	0.693
Chi2(*2*)	4.965	1.078	1.164	0.187	5.950	5.544	0.947	1.925	0.468	8.029
*p*−value	0.084	0.583	0.559	0.911	0.051	0.063	0.623	0.382	0.791	0.018
RMSEA	0.131	0.000	0.000	0.000	0.159	0.144	0.000	0.000	0.000	0.197
CFI	0.895	1.000	1.000	1.000	0.952	0.936	1.000	1.000	1.000	0.946
TLI	0.631	1.150	1.126	1.172	0.834	0.650	1.126	1.005	1.104	0.703
SRMR	0.060	0.018	0.023	0.008	0.046	0.046	0.012	0.024	0.011	0.038

* *p* < 0.1, ** *p* < 0.05, *** *p* < 0.01. INT = intention, PBC = perceived behavioral control, HBT = habit, PN = perceived need, ATT = attitudes, and SN = subjective norm. For the sake of space, the standard errors and *p*-values are not listed in the table but can be obtained from the authors.

**Table 6:** Indirect effects of TPB constructs on frequency of consuming meat, eggs, dairy, fish, and fruits of standard and extended TPB models.

	Standardized Coeff. (Standard TPB)					Standardized Coeff. (Extended TPB)				
	Meat	Eggs	Dairy	Fish	Fruit	Meat	Eggs	Dairy	Fish	Fruit
PBC	0.010	−0.002	−0.001	0.010	0.049	−0.012	0.004	0.010	0.015	0.011
ATT	0.039	0.083 **	0.006	0.041 **	0.091 **	0.051	0.206 *	−0.022	0.007	0.031
SN	0.016	0.021	0.001	0.030 *	0.119 **	−0.010	0.061	0.006	0.005	0.043
HBT						0.370	0.147	0.157	0.320 ***	0.380
PN						−0.093	0.472 *	0.247	0.164	0.768 *

* *p* < 0.1, ** *p* < 0.05, *** *p* < 0.01. INT = intention, PBC = perceived behavioral control, HBT = habit, PN = perceived need, ATT = attitudes, and SN = subjective norm.

## Appendix A

**Table A1:** Descriptive statistics (means, standard deviations) of the TPB construct items for predicting consumption frequency of meat, eggs, dairy, fish, and fruit.

	Meat (*n* = 86)		Egg (*n* = 92)		Dairy (*n* = 85)		Fish (*n* = 83)		Fruit (*n* = 78)	
	M	SD	M	SD	M	SD	M	SD	M	SD
**Frequency**	5.71	1.70	1.47	1.71	0.93	2.18	0.42	1.05	3.49	2.79
**Intention**	4.12	0.67	3.39	0.99	2.41	1.03	2.55	1.03	3.82	0.90
**ATT**	15.92	2.65	14.79	2.06	13.29	3.06	14.44	3.00	16.01	3.57
att1	16.50	3.72	16.54	3.79	15.28	3.64	15.45	3.77	17.64	4.30
att1_str	3.83	0.67	3.91	0.60	3.59	0.74	3.73	0.68	4.09	0.67
att1_imp	4.31	0.54	4.21	0.55	4.26	0.52	4.11	0.44	4.27	0.53
att2	15.41	3.47	15.72	2.89	14.93	3.63	15.07	3.23	16.35	3.87
att2_str	3.86	0.62	3.92	0.47	3.76	0.72	3.81	0.59	3.99	0.69
att2_imp	3.97	0.47	4.00	0.53	3.95	0.55	3.94	0.45	4.08	0.53
att3	16.08	3.30	14.16	3.31	12.20	4.04	13.76	3.79	15.44	4.11
att3_str	4.05	0.59	3.71	0.62	3.13	0.96	3.63	0.73	3.88	0.66
att3_imp	3.95	0.43	3.83	0.62	3.93	0.57	3.81	0.67	3.94	0.57
att4	15.71	3.77	12.74	3.55	10.74	4.23	13.49	4.06	14.62	4.54
att4_str	4.02	0.61	3.51	0.76	2.89	0.86	3.60	0.83	3.74	0.75
att4_imp	3.88	0.56	3.65	0.67	3.66	0.70	3.75	0.64	3.85	0.67
**SN**	15.26	3.07	14.49	3.19	13.48	3.57	13.16	3.91	16.07	3.94
sn1	14.79	4.47	13.57	4.04	12.33	4.59	12.53	4.62	15.41	4.83
sn1_str	3.59	0.85	3.39	0.82	3.04	0.98	3.17	0.91	3.81	0.81
sn1_imp	4.09	0.63	4.00	0.59	4.04	0.63	3.89	0.62	4.00	0.68
sn2	13.38	5.37	13.29	5.36	12.68	5.31	12.67	5.09	15.73	5.37
sn2_str	3.26	1.05	3.21	1.03	3.06	1.08	3.16	0.98	3.76	0.93
sn2_imp	4.09	0.64	4.10	0.65	4.11	0.66	3.96	0.65	4.14	0.66
sn3	16.42	3.85	15.63	3.56	14.71	4.53	13.78	4.69	16.58	4.13
sn3_str	4.00	0.59	3.82	0.61	3.48	0.87	3.45	0.90	3.97	0.62
sn3_imp	4.07	0.50	4.07	0.44	4.19	0.48	3.94	0.59	4.13	0.47
sn4	16.42	3.85	15.63	3.56	14.71	4.53	13.78	4.69	16.58	4.13
sn4_str	4.02	0.59	3.79	0.73	3.41	0.90	3.47	0.89	3.99	0.67
sn4_imp	4.05	0.55	4.03	0.46	4.11	0.49	3.88	0.55	4.10	0.47
**PBC**	14.88	3.45	14.23	3.32	13.80	3.89	13.58	3.64	15.01	3.83
pbc1	14.76	3.97	14.21	3.77	13.72	4.37	13.29	4.32	14.87	4.04
pbc1_str	3.78	0.76	3.83	0.81	3.35	0.98	3.52	1.02	3.74	0.80
pbc1_imp	3.93	0.78	3.76	0.79	4.11	0.62	3.84	0.79	3.97	0.70
pbc2	15.00	3.93	14.26	4.24	13.88	4.95	13.87	4.39	15.15	4.93
pbc2_str	3.95	0.63	3.88	0.71	3.56	0.97	3.59	0.95	3.83	0.83
pbc2_imp	3.78	0.68	3.66	0.76	3.87	0.78	3.89	0.64	3.91	0.72
**PN**	4.08	0.60	3.51	0.78	2.88	0.96	2.96	0.96	3.86	0.88
**HBT**	4.27	0.64	3.13	1.04	2.14	1.03	2.39	0.99	3.47	1.14

INT = intention, PBC = perceived behavioral control, HBT = habit, PN = perceived need, ATT = attitudes, and SN = subjective norm. “_str” means strength of belief, “_imp” means evaluation of importance. att1 = healthy, att2 = nutritious, att3 = tasty, att4 = satisfactory; sn1 = family’s opinion, sn2 = doctor’s opinion, sn3 = give family a healthy diet, sn4 = give family a nutritious diet; pbc1 = affordability, pbc2 = accessibility.

**Table A2:** Correlation between TPB constructs, perceived need, and habit for meat consumption (*n* = 86).

	Frequency	Intention	ATT	SN	PBC	HBT
Intention	0.2563 **					
ATT	0.0081	0.4274 ***				
SN	−0.0581	0.3122 ***	0.4909 ***			
PBC	0.2787 ***	0.2625 **	0.4314 *	0.1518		
HBT	0.2127 **	0.4528 ***	0.2671 **	0.4386 ***	0.3851 ***	
PN	0.1702 ***	0.5821 ***	0.2639 **	0.3534 ***	0.3668 ***	0.5874 ***

* *p* < 0.1, ** *p* < 0.05, *** *p* < 0.01. INT = intention, PBC = perceived behavioral control, HBT = habit, PN = perceived need, ATT = attitudes, and SN = subjective norm.

**Table A3:** Correlation between TPB constructs, perceived need, and habit for egg consumption (*n* = 92).

	Frequency	Intention	ATT	SN	PBC	HBT
Intention	0.3032 ***					
ATT	0.2090 **	0.4066 ***				
SN	0.0713	0.3092 ***	0.5104 ***			
PBC	0.1048	0.1300	0.3705 ***	0.1054		
HBT	0.2068 **	0.4453 ***	0.1830 *	0.2264 **	−0.044	
PN	0.2562 ***	0.5334 ***	0.3430 ***	0.2651 **	0.0491	0.5147 ***

* *p* < 0.1, ** *p* < 0.05, *** *p* < 0.01. INT = intention, PBC = perceived behavioral control, HBT = habit, PN = perceived need, ATT = attitudes, and SN = subjective norm.

**Table A4:** Correlation between TPB constructs, perceived need, and habit for dairy consumption (*n* = 85).

	Frequency	Intention	ATT	SN	PBC	HBT
Intention	0.0305					
ATT	−0.0648	0.4297 ***				
SN	0.0894	0.2719 **	0.4405 ***			
PBC	0.3457 ***	0.0230	0.0410	0.2196 **		
HBT	0.1695	0.7355 ***	0.3649 ***	0.3101 ***	0.1520	
PN	0.3265 ***	0.5914 ***	0.3937 ***	0.3564 ***	0.0752	0.5028 ***

* *p* < 0.1, ** *p* < 0.05, *** *p* < 0.01. INT = intention, PBC = perceived behavioral control, HBT = habit, PN = perceived need, ATT = attitudes, and SN = subjective norm.

**Table A5:** Correlation between TPB constructs, perceived need, and habit for fish consumption (*n* = 83).

	Frequency	Intention	ATT	SN	PBC	HBT
Intention	0.4307 ***					
ATT	0.1958 *	0.4477 ***				
SN	0.1611	0.4549 ***	0.5908 ***			
PBC	0.0981	0.2848 ***	0.2262 **	0.3229 ***		
HBT	0.3952 ***	0.6180 ***	0.3326 ***	0.3701 ***	0.1477	
PN	0.2277 ***	0.6347 ***	0.4601 ***	0.4155 ***	0.0253	0.5069 ***

* *p* < 0.1, ** *p* < 0.05, *** *p* < 0.01. INT = intention, PBC = perceived behavioral control, HBT = habit, PN = perceived need, ATT = attitudes, and SN = subjective norm.

**Table A6:** Correlation between TPB constructs, perceived need, and habit for fruit consumption (*n* = 78).

	Frequency	Intention	ATT	SN	PBC	HBT
Intention	0.4997 ***					
ATT	0.1639	0.6267 ***				
SN	0.3367 ***	0.6844 ***	0.6616 ***			
PBC	0.3788 ***	0.5505 ***	0.5130 ***	0.6167 ***		
HBT	0.4306 ***	0.6796 ***	0.4379 ***	0.5491 ***	0.4672 ***	
PN	0.4645 ***	0.7199 ***	0.5880 ***	0.5652 ***	0.4928 ***	0.6791 ***

* *p* < 0.1, ** *p* < 0.05, *** *p* < 0.01. INT = intention, PBC = perceived behavioral control, HBT = habit, PN = perceived need, ATT = attitudes, and SN = subjective norm.
